# Metabolic flexibility ensures proper neuronal network function in moderate neuroinflammation

**DOI:** 10.1038/s41598-024-64872-1

**Published:** 2024-06-22

**Authors:** Bruno Chausse, Nikolai Malorny, Andrea Lewen, Gernot Poschet, Nikolaus Berndt, Oliver Kann

**Affiliations:** 1 Institute of Physiology and Pathophysiology, Heidelberg University, Im Neuenheimer Feld 326, 69120 Heidelberg, Germany; 2 Metabolomics Core Technology Platform, Centre for Organismal Studies, Heidelberg University, 69120 Heidelberg, Germany; 3 Department of Molecular Toxicology, German Institute of Human Nutrition Potsdam-Rehbruecke (DIfE), 14558 Nuthetal, Germany; 4 Institute of Computer-Assisted Cardiovascular Medicine, Deutsches Herzzentrum der Charité (DHZC), 13353 Berlin, Germany; 5 Charité – Universitätsmedizin Berlin, corporate member of Freie Universität Berlin and Humboldt-Universität zu Berlin, 10117 Berlin, Germany; 6 Interdisciplinary Center for Neurosciences (IZN), Heidelberg University, 69120 Heidelberg, Germany; 7 Present Address: MEDISS Doctoral Program, INF 110, Heidelberg University, 69120 Heidelberg, Germany

**Keywords:** Glycolysis, Immunometabolism, Lactate oxidation, Microglia, Neuronal oscillations, Neuroimmunology, Microglia

## Abstract

Microglia, brain-resident macrophages, can acquire distinct functional phenotypes, which are supported by differential reprogramming of cell metabolism. These adaptations include remodeling in glycolytic and mitochondrial metabolic fluxes, potentially altering energy substrate availability at the tissue level. This phenomenon may be highly relevant in the brain, where metabolism must be precisely regulated to maintain appropriate neuronal excitability and synaptic transmission. Direct evidence that microglia can impact on neuronal energy metabolism has been widely lacking, however. Combining molecular profiling, electrophysiology, oxygen microsensor recordings and mathematical modeling, we investigated microglia-mediated disturbances in brain energetics during neuroinflammation. Our results suggest that proinflammatory microglia showing enhanced nitric oxide release and decreased CX3CR1 expression transiently increase the tissue lactate/glucose ratio that depends on transcriptional reprogramming in microglia, not in neurons. In this condition, neuronal network activity such as gamma oscillations (30–70 Hz) can be fueled by increased ATP production in mitochondria, which is reflected by elevated oxygen consumption. During dysregulated inflammation, high energy demand and low glucose availability can be boundary conditions for neuronal metabolic fitness as revealed by kinetic modeling of single neuron energetics. Collectively, these findings indicate that metabolic flexibility protects neuronal network function against alterations in local substrate availability during moderate neuroinflammation.

## Introduction

Brain metabolism must be precisely regulated to maintain proper neuronal function^1,2^. During increased neuronal activity, for instance, dynamic adjustments in local cerebral blood flow are essential to ensure adequate supply with oxygen and nutrients such as glucose^3,4^. Neuronal function is also dependent on a complex metabolic interaction with astrocytes, which includes the exchange of nutrients such as lactate^5^, glutamine/glutamate^6^, and lipids^7,8^. The metabolic utilization of lactate, for example, requires cellular uptake through H^+^-coupled monocarboxylate transporters (MCTs), conversion back to pyruvate as well as subsequent oxidation in mitochondria that requires oxygen to produce ATP^9^. Although the metabolic coupling between neurons and astrocytes has been intensively investigated, the roles of other glial cells in brain energy homeostasis have yet to be explored^9^.

An intriguing example is microglia, brain-resident immune cells, which integrate molecular signals to orchestrate responses against threats to tissue integrity^10^. Upon identifying cellular damage and/or pathogen molecular patterns, these cells undergo complete functional remodeling that includes a differential reprogramming of cell metabolism^11,12^. Indeed, reactive states of microglia are supported by defined metabolic adaptations such as remodeling in glycolytic and mitochondrial metabolic fluxes^13,14^, triggering of a lipid biosynthetic program^15,16^, and upregulation in oxidant production^17^.

Although microglial function has been often explored from an immunological perspective, recent evidence suggests that these cells may also influence brain metabolism. Disruption of lactate transport in microglia, for instance, alters synapse maturation leading to defects in brain development and adult behavior in mice^18^. Microglia also interact with astrocytes in the juxtavascular space, where they can participate in the regulation of blood–brain barrier integrity and cerebral blood flow^19^. Importantly, microglia can be more glycolytic and present higher glucose uptake than neurons and glial cells in healthy^20^ and diseased brain tissue^21^, a phenotype also observed in induced pluripotent stem cells (iPSC)-derived human microglia^22^. Indeed, microglia mediate a local increase in glucose consumption and lactate production in inflamed cortical tissue, further suggesting that these cells may alter energy substrate availability in the brain^13,23^. This phenomenon may be relevant for high order neuronal functions such as network oscillations, which arise from precise synaptic interactions between principal neurons and interneurons and are exquisitely sensitive to alterations in metabolism and redox balance^2,24–27^. The impacts of microglia-mediated changes on energy substrate availability in the brain parenchyma have been ignored, however.

Combining molecular profiling, electrophysiology, RNA-seq analyses, and metabolic mathematical modeling, we show that a transcriptional program in microglia induces a transient change in the lactate/glucose ratio in inflamed cortical tissue, whereas neuronal network activity can be still sufficiently fueled by increased ATP production in mitochondria. Notably, high energy demand and low glucose availability might limit neuronal energy homeostasis, including proper network function during dysregulated inflammation. These findings suggest that microglia can affect neuronal substrate utilization, providing new insights into metabolic interactions between cells in the brain parenchyma.

## Results

### Inflammation transiently modulates energy substrate availability in the brain

**Figure 1:**
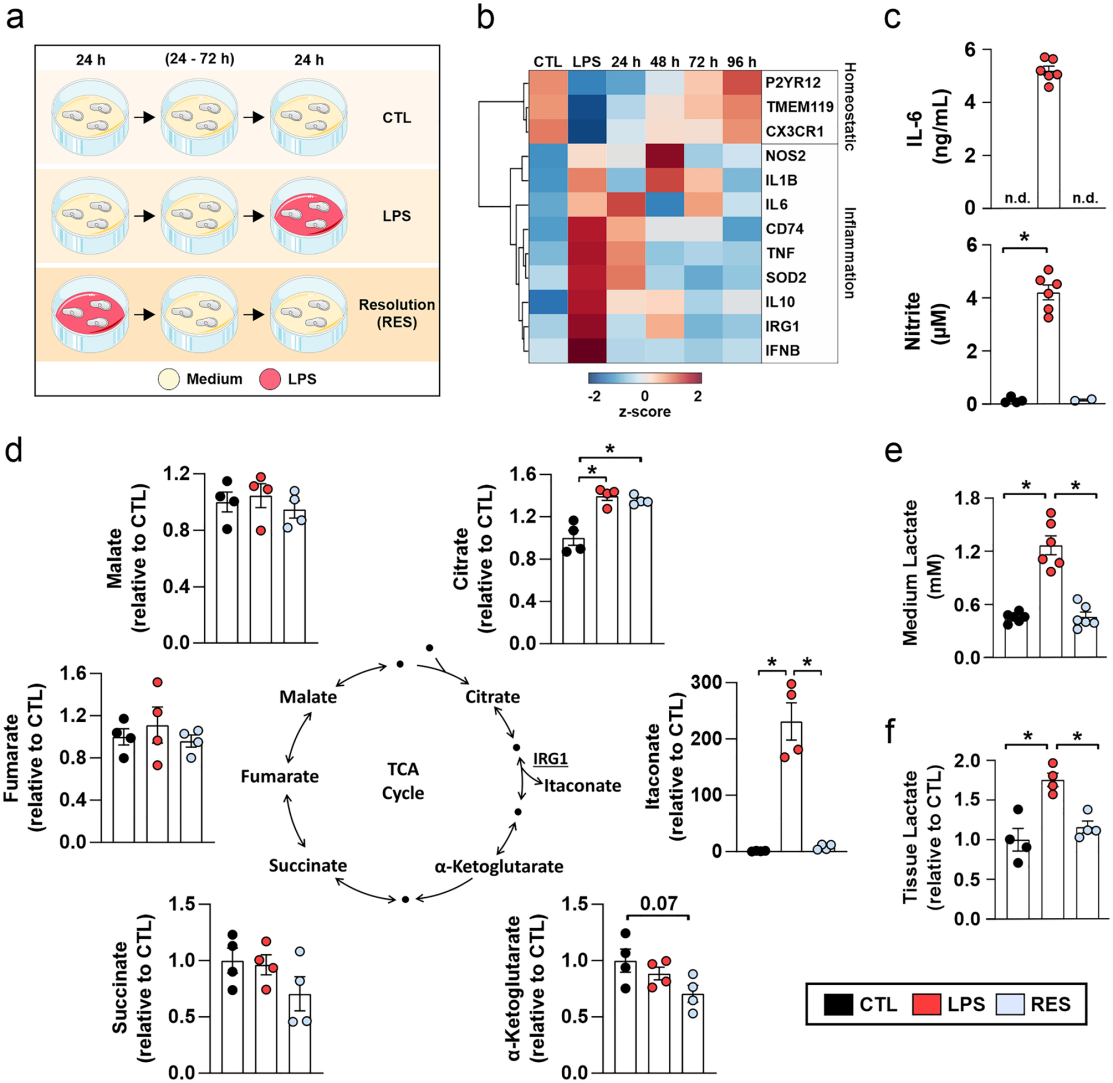
Moderate inflammation transiently alters brain energy metabolism in situ. (**a**) Slice cultures were exposed to medium (Control group - CTL), LPS (100 ng/mL) in the last 24 h before tissue processing (LPS group) or LPS for 24 h followed by incubation with LPS-free medium for additional 24, 48, 72 or 96 h (Resolution - RES). Medium from all groups was changed daily. In the bar graphics (**c–f**), RES refers to 96 h after LPS removal. (**b**) Hierarchical clustering and heatmap depicting differential expression of inflammatory and microglial homeostatic genes as measured by RT-PCR. The color code represents then the number of standard deviations a sample is from the mean of expression distribution in the row. Note that the LPS response is resolved after 96 h. (**c**) IL-6 and nitrite content in culture supernatants. Note that nitrite was not detected in one sample of the RES group and statistics were performed by comparing the CTL and LPS groups only. (**d**) Metabolite content in whole tissue homogenates. Values are normalized by the content in the control group. (**e**) Lactate content in culture supernatants. (**f**) Lactate content in whole tissue homogenates. Values represent averages ± SEM and were compared using unpaired t test (**c**) or one-way ANOVA followed by Tukey’s post hoc test (**d-f**). *P < 0.05. For n/N membranes or membrane pools/animals: (**b**) 3 pools/6, (**c**) 6/6, (**d**) 4 pools/8, (**e**) 6/6, (**f**) 4 pools/8.

To explore the effects of transient inflammation on brain metabolism, we employed male rat organotypic hippocampal slice cultures that feature well-preserved brain cytoarchitecture and natural neuron-glia interactions, and express high order neuronal functions such as neuronal network oscillations in the gamma-band (30–70 Hz)^28–31^. Only male rats were employed to avoid the impacts of sex-dependent variability in neuronal and microglial biology^32^. Slice cultures were treated with medium (CTL), 24 h lipopolysaccharide (LPS) acutely before sample processing or 24 h LPS followed by 24, 48, 72 or 96 h LPS-free medium (resolution—RES) (Fig. 1a). LPS upregulated the expression of inflammation- and stress-related genes in parallel to a downregulation of microglial homeostatic genes, a hallmark feature of proinflammatory microglia (Fig. 1b)^33^. This phenotype was fully reversed after 96 h of LPS removal, the time point hereafter referred to as resolution (RES). Importantly, transient inflammation was also observed when measuring inflammatory mediator content in culture media (Fig. 1c).

We next employed targeted metabolomics to uncover adaptations in brain metabolism during transient inflammation, which revealed significant alterations in Krebs cycle metabolites at the tissue level. Inflammation onset featured an accumulation in citrate that resulted in decreased α-ketoglutarate/citrate ratio (Fig. 1d; Supplementary Fig. 1a), suggesting a reduced carbon flow through the isocitrate dehydrogenase reaction. Indeed, the content of the immunometabolite itaconate (Fig. 1d) as well as the expression of IRG1 (Fig. 1b), the gene encoding the enzyme producing itaconate, were strongly induced by LPS, and might represent an important source of carbon deviation from the Krebs cycle^13,34,35^. Since LPS already induces metabolic alterations after a few hours in microglial monocultures^13,22,36^, we next tested if changes in metabolism also occur early at the tissue level. Although inflammatory markers were identified in slice culture supernatants after 6 h, metabolic changes were first seen after 8 h of LPS treatment (Supplementary Fig. 1b, c). Indeed, metabolomics experiments confirmed that Krebs cycle carbon flow was already changed at this time point (Supplementary Fig. 1d–g), suggesting that brain metabolism is quickly remodeled during inflammation. Interestingly, while IRG1 and itaconate content returned to control levels, the alterations in citrate and in the α-ketoglutarate/citrate ratio persisted after inflammation resolution (Fig. 1b, d; Supplementary Fig. 1a).

We asked next if transient inflammation could alter the availability and usage of glucose and lactate in the brain parenchyma. Inflammation onset featured an increase in lactate content in the culture medium (Fig. 1e) and in the tissue (Fig. 1f). Metabolomics experiments revealed that tissue lactate was already higher after 8 h LPS (Supplementary Fig. 1h), confirming that inflammation induces a fast remodeling in brain glucose utilization. Strikingly, lactate content returned to control levels after resolution (Fig. 1e, f), an indicative that inflammation transiently modulates energy substrate availability in brain tissue.

### A transcriptional reprogramming in microglia drives brain lactate production during inflammation

**Figure 2:**
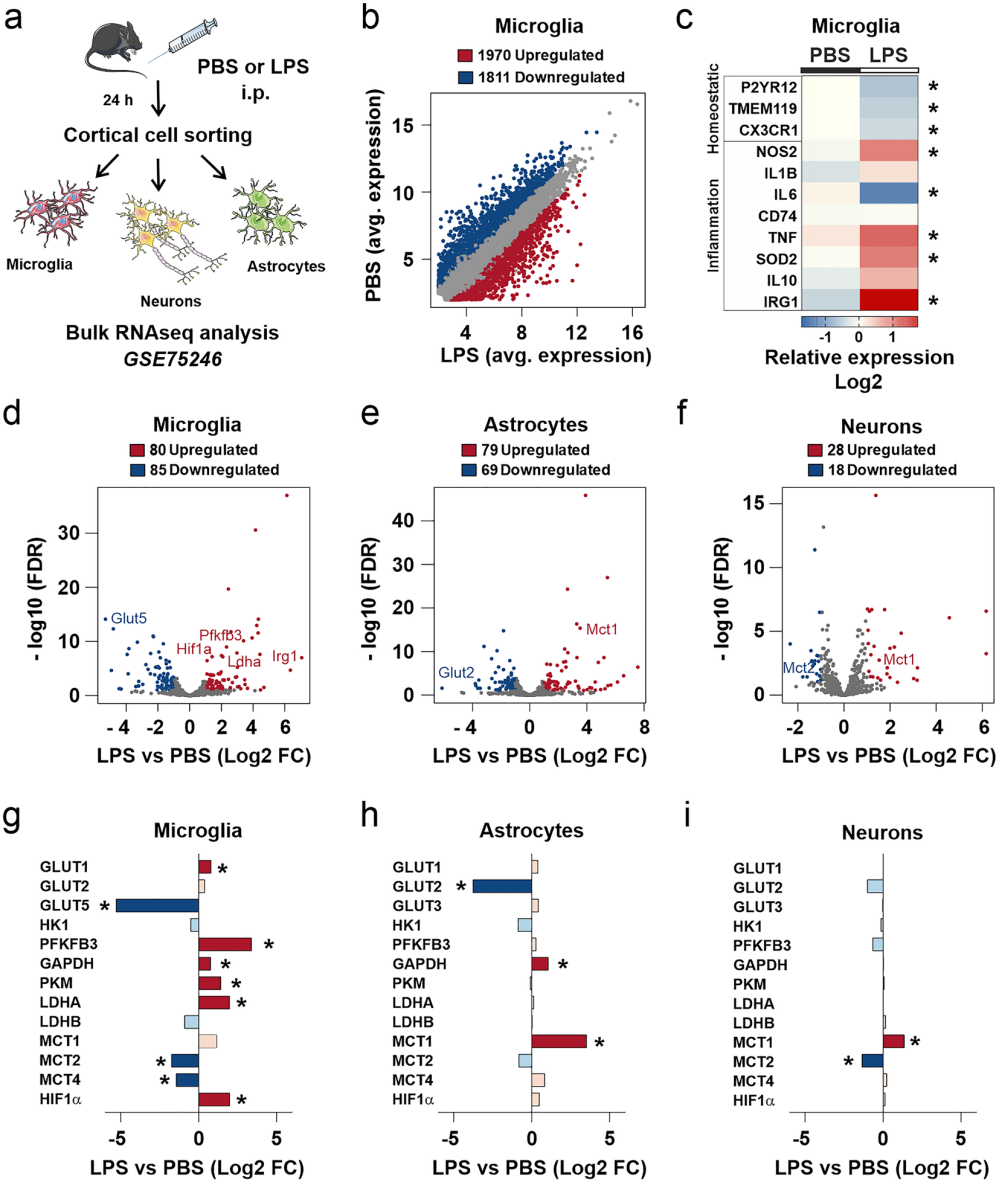
A transcriptional reprogramming in microglia drives the alterations in brain metabolism during inflammation in vivo. (**a**) The dataset generated by Srinivasan et al. 2016 (GSE75246) was employed to explore cell-specific changes in metabolic genes during inflammation. In their experimental design, microglia, neurons and astrocytes were separated by cell sorting 24 h after intraperitoneal LPS injection (ipLPS) and then characterized by bulk RNAseq analyses. (**b**) Scatter plot showing differentially expressed genes in ipLPS versus ipPBS (intraperitoneal PBS injection) microglia (fold change > 2; false discovery rate cutoff: 0.1; see Methods). Note that intraperitoneal LPS effectively induces a transcriptional reprograming in microglia. (**c**) Differential expression of inflammatory and microglial homeostatic genes in ipLPS versus ipPBS microglia. Heatmap color code represents medians of the relative gene expression (Log2). Note the overlap between genes changing in ipLPS microglia and in the direct exposure of slice cultures to LPS (Fig. 1b). To evaluate cell-specific changes in metabolic genes during inflammation, differential expression analyses were performed using a list containing 789 genes listed in the GO terms carbohydrate metabolic processes, ATP processes, TCA processes plus individually added metabolic regulators and transporters (fold change > 2; false discovery rate cutoff: 0.1; see Methods). Volcano plots showing differentially expressed metabolic genes in ipLPS versus ipPBS (**d**) microglia, (**e**) astrocytes and (**f**) neurons. Differential expression of glycolytic genes in ipLPS versus ipPBS (**g**) microglia, (**h**) astrocytes and (**i**) neurons. *Padj < 0.05. N/group: For microglia and neurons—5 animals/treatment group, for astrocytes—4 animals/treatment group. FC—Fold Change. FDR—False Discovery Rate.

To investigate if the changes in glucose metabolism were supported by transcriptional alterations in metabolic genes, we first measured the expression of the glucose transporter GLUT1 and the lactate transporter MCT1 in slice cultures treated according to the paradigm depicted in Fig. 1a. However, there were no alterations in expression of these genes at the tissue level (Supplementary Fig. 1i, j). We next assessed if changes in brain metabolism were driven by cell-specific transcriptional programs by exploring a gene expression dataset generated by Srinivasan et al.^37^—accession code GSE75246. In their in vivo study, microglia, neurons and astrocytes were separated by cell sorting 24 h after intraperitoneal LPS injection (ipLPS) in male mice and then characterized by bulk RNAseq analyses (Fig. 2a). We first explored global alterations in microglial gene expression and confirmed that ipLPS efficiently remodeled microglial transcriptome in the brain (Fig. 2b). There was an overlap between genes changing in ipLPS microglia (Fig. 2c) and in slice cultures directly exposed to LPS (Fig. 1b), suggesting that the inflammatory states in the two models are comparable^38^. Although the astrocyte transcriptome was also remodeled and markers of pan-reactive astrocytes were broadly upregulated by ipLPS (Supplementary Fig. 2a, b)^39^, the inflammatory genes assessed in slice cultures (Fig. 1b) were almost not altered in these cells (Supplementary Fig. 2b). Notably, the LPS-induced release of inflammatory mediators was abrogated in microglia-depleted slice cultures, further indicating that most inflammatory signals originate from microglia (Supplementary Fig. 3a–d).

We next focused on changes in brain metabolism by performing differential expression analyses on genes listed in the following gene ontology (GO) terms: carbohydrate metabolic processes, ATP processes, and TCA processes. Additional metabolic regulators and transporters were individually added (complete list in supplementary information). Microglia were the most sensitive population to changes in metabolic genes followed by astrocytes and then neurons (Fig. 2d–f). Despite the array of evidence indicating metabolic coupling between cells in the brain parenchyma, only a few genes were altered in parallel in microglia, neurons and astrocytes and their identity alone cannot explain the alterations in tissue glucose metabolism (Supplementary Fig. 4a–c; Tables 6, 7 and 8 in supplementary information). Indeed, there was co-modulation only in 13 (of 198), 36 (of 282) and 20 (of 176) genes in the pair microglia-neurons, microglia-astrocytes and astrocytes-neurons, respectively. A focused analysis on the expression of glycolysis-related genes suggested, however, that glucose usage and lactate production might be enhanced during inflammation by a specific transcriptional program in microglia (Fig. 2g–i). Interestingly, LPS-induced enrichment in lactate content was not present in microglia-depleted slice cultures (Supplementary Fig. 3e), further supporting the hypothesis that microglia drive metabolic changes in the inflamed brain. Similar alterations in the glycolytic gene expression were also found in datasets of engrafted human microglia (Supplementary Fig. 4d), amyotrophic lateral sclerosis (Supplementary Fig. 4e), and Alzheimer’s disease models (Supplementary Fig. 4f), suggesting that enriched glycolytic flow might be a signature of activated microglia.

### Metabolic flexibility ensures neuronal network function under modified lactate/glucose ratios

**Figure 3:**
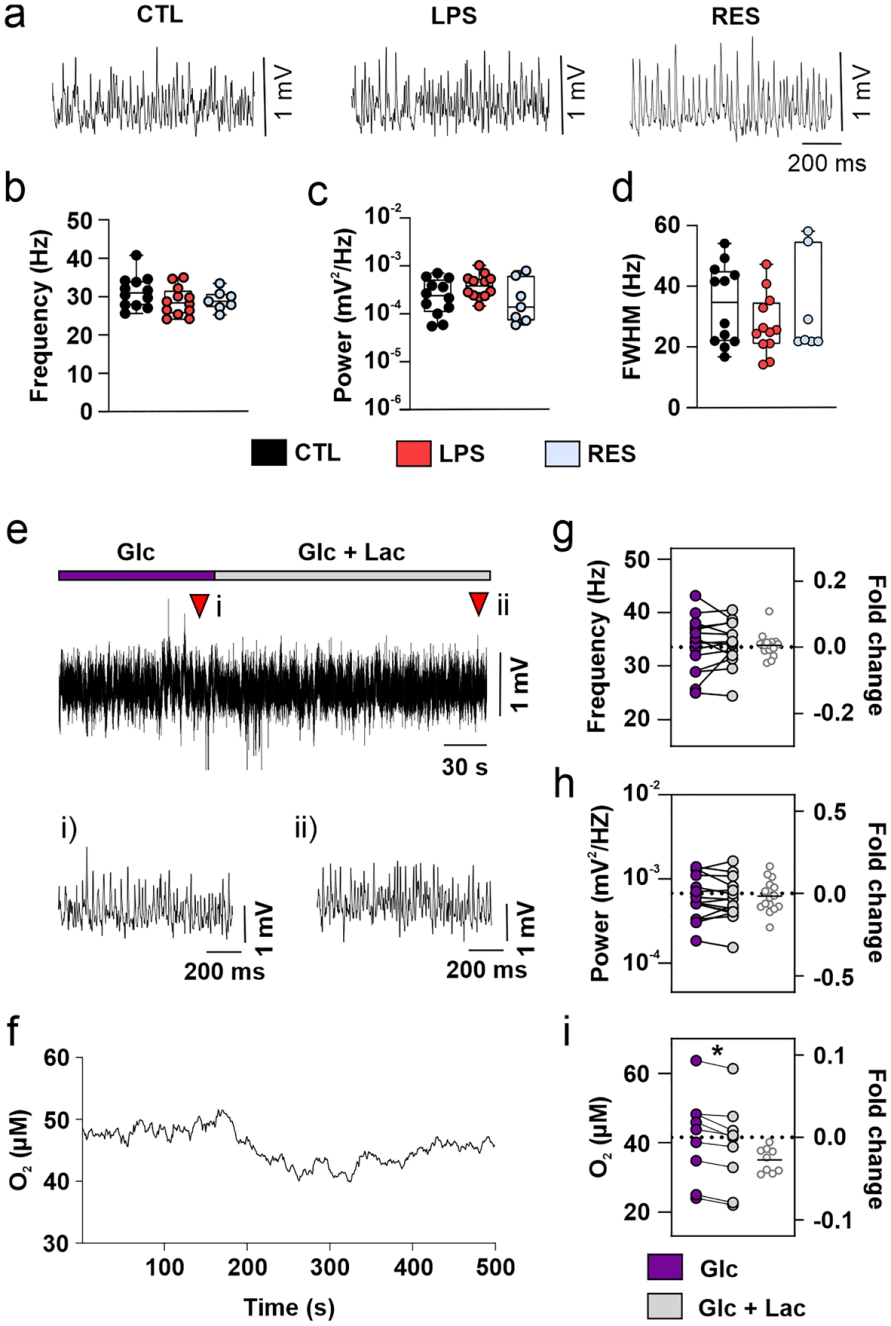
Metabolic flexibility ensures neuronal network function under higher lactate/glucose ratios. Slice cultures from control (CTL), LPS and resolution (RES) groups were placed in the interface chamber for local field potential recordings. Gamma oscillations (30–70 Hz) were induced by continuous application of the cholinergic agonist carbachol, and recordings were carried out in stratum pyramidale of the hippocampal CA3 region. (**a**) Sample traces of slices expressing gamma oscillations. Gamma oscillation properties: (**b**) peak frequency, (**c**) peak power and (**d**) full width at half-maximum (FWHM). In a second experimental paradigm, gamma oscillations were induced in untreated slice cultures by continuous application of carbachol in ACSF containing 5 mM glucose. After 30 min, ACSF containing 5 mM glucose plus 2 mM lactate was added to the interface chamber. An oxygen sensor was placed in the core of the slices to record simultaneous changes in the network activity and oxygen consumption. (**e**) Sample traces of gamma oscillations before and after the ACSF change and (**f**) parallel changes in oxygen concentration in the core of the same slice. Red triangles show the time points where the representative traces (i) and (ii) were sampled. Gamma oscillation properties: (**g**) peak frequency and (**h**) peak power. (**i**) Oxygen concentration in slice cores. In (**b-d**), values represent medians and interquartile range and were compared using one-way ANOVA followed by Tukey’s post hoc test (**b**) or Kruskal–Wallis test with Dunn’s post hoc test (**c, d**). The whiskers indicate minimum and maximum of data. In **g-i**, values in the left side (purple and gray circles) represent measurements in individual slices before and after the ACSF change and were compared using paired t test. Values in the right side (small white circles) represent the fold change of glucose plus lactate vs glucose only. *P < 0.05. For n/N slices/animals: (**b**) 7–12/3–4, (**c**) 7–12/3–4, (**d**) 7–12/3–4, (**g**) 15/6, (**h**) 15/6, (**i**) 9/6.

To address if metabolic changes in inflamed cortical tissue affect neuronal function, we assessed neuronal network oscillations in the gamma-band (30–70 Hz) in slice cultures treated according to the paradigm depicted in Fig. 1a. Gamma oscillations arise from precise synaptic interactions between excitatory pyramidal cells and inhibitory interneurons and underlie higher cognitive functions, such as sensory perception and memory formation^2,40^. These rhythmic activities are readily disturbed by metabolic and redox stress and provide a sensitive readout for physiological neural network function^24,27,41^. Notably, the properties of gamma oscillations were unchanged during inflammation onset (LPS) or resolution (RES) (Fig. 3a), despite the molecular and metabolic changes in the tissue (see Fig. 1). Indeed, oscillation frequency (Fig. 3b) as well as peak power (Fig. 3c) and full width at half-maximum (FWHM) (Fig. 3d), which primarily reflect number and synchrony of postsynaptic currents, were similar in all treatment groups. Prolonged exposure to LPS at high concentration (72 h, 10 µg/ml), however, associated with moderate changes in gamma oscillation properties^30^.

Since transcriptional changes in microglia were related to increased lactate content at the tissue level (Figs. 1 and 2), we hypothesized that neurons could cope with the altered metabolic milieu by modulating energy substrate utilization during inflammation. To explore this hypothesis, we designed a proof-of-concept experiment in untreated slice cultures, in which gamma oscillations were characterized in the presence of artificial cerebrospinal fluid (ACSF) containing glucose only. To simulate a microglia-induced increase in the lactate/glucose (Lac/Glc) ratio, gamma oscillations were then characterized in the presence of glucose plus lactate (Fig. 3e). In parallel, we monitored real-time adaptations in oxidative energy metabolism by measuring changes in the local oxygen concentration using an oxygen microsensor placed at a depth of 80–96 µm in the tissue (slice core). Note that oxygen concentration in slice core is a product of oxygen diffusion through the tissue and its consumption by adjacent cells^31,42^. Since gas supply is clamped in the interface recording chamber (rapid exchange of the gas mixture containing 20% oxygen fraction), a decrease in oxygen concentration provides a proxy for increased oxygen consumption in the tissue (Fig. 3f). Although gamma oscillation frequency (Fig. 3g) and peak power (Fig. 3h) were not affected by changes in energy substrate availability, the oxygen concentration was lower in the presence of glucose plus lactate (Fig. 3i). These data suggest that enhanced lactate oxidation in mitochondria contributes to proper neuronal network function at increased Lac/Glc ratios.

### Energy demand and glucose availability limit neuronal energy fitness during inflammation

To explore the boundary conditions for changes in Lac/Glc ratio that active neurons can tolerate, we next employed a single neuron kinetic model to estimate glucose uptake and lactate release/uptake in different energy demands and extracellular Lac/Glc ratios^43^. Extracellular oxygen was kept constant at saturating levels of 60 mmHg and intracellular ATP was employed as a proxy for neuronal energy fitness. Note that neuronal ATP is strictly maintained in a millimolar range and concentrations below 1 mM were therefore considered indicative of energy deficit^44,45^.

**Figure 4:**
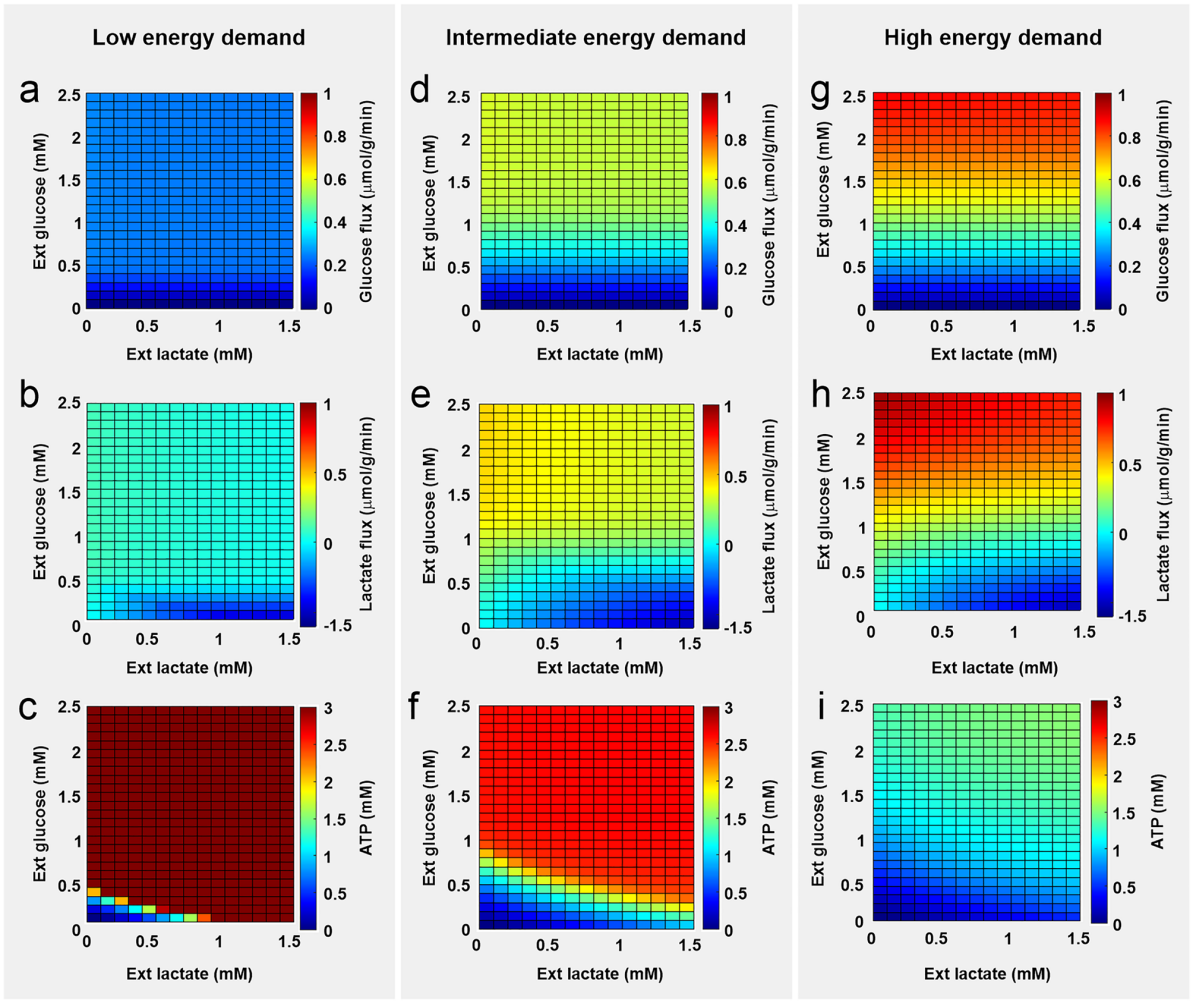
High-energy demand and low glucose availability limit neuronal energy fitness during inflammation. A single-neuron kinetic model was employed to estimate glucose influx, lactate outflux/influx and ATP concentration during low (**a-c**), intermediate (**d-f**) and high (**g-i**) energy demands in different extracellular lactate/glucose ratios. For lactate fluxes (**b**, **e**, **h**), negative values indicate lactate uptake and positive values lactate release. The model comprises the conversion of glucose to pyruvate/lactate in glycolysis; shuttles of electrons (NAD-bound hydrogen) between the cytosol and the mitochondrial matrix; the TCA cycle; the mitochondrial respiratory chain, proton gradient and mitochondrial membrane potential; the rate of the adenine nucleotide exchanger exchanging mitochondrial ATP against cytosolic ADP; the exchange of cations and anions across the inner mitochondrial membrane; exchange of lactate with the external space by the monocarboxylate transporter, MCT2; and, the reversible intracellular conversion of lactate into pyruvate by the lactate dehydrogenases LDHA and LDHB (more details in Material and Methods).

At low energy demand, glucose uptake (Fig. 4a) and lactate release (Fig. 4b) remained low and constant in almost all tested Lac/Glc ratios. Lactate uptake was only observed when a raise in the Lac/Glc ratio was induced by pronounced reduction in glucose availability (Fig. 4b). Interestingly, neurons were able to maintain energy fitness in the absence of glucose when extracellular lactate content was elevated, a state that might be dependent on proper oxygen supply and ATP production in mitochondria (Fig. 4c)^4^. Indeed, we have shown that neuronal energy metabolism can be reliably adapted when lactate replaces glucose during network rhythms with lower energy expenditure such as sharp wave-ripples^24^. At intermediate energy demand, neuronal energy requirements were fulfilled by enhanced glucose uptake (Fig. 4d) and lactate release (Fig. 4e) in almost all Lac/Glc ratios. Although the reduction in glucose availability was compensated by lactate uptake, neuronal energy fitness was only maintained when extracellular glucose was higher than approx. 0.25 mM (Fig. 4f). Below this threshold, intracellular ATP collapsed even under high lactate availability (Fig. 4f). Neuronal dependence on extracellular glucose was even more evident at a higher energy demand (Fig. 4g–i). In this state, neurons only maintained energy fitness when glucose levels were higher than 1.5 mM. These data suggest that high energy demand and low glucose availability might be the boundaries for neuronal energy fitness during changes in Lac/Glc ratio in the inflamed brain.

## Discussion

Glucose is the main substrate imported from the bloodstream to fuel brain energy processes^46^. If these processes are supported by complete oxidation of glucose or also include lactate shuttling between brain cells is a matter of heated debate^9,47,48^. A new complexity level was recently added to this discussion by the observation that microglia can present higher glycolytic rates than the neuropil^20^ and that activated microglia can drive brain glucose uptake in neurodegenerative diseases^21^. Here, we show that a transcriptional program in microglia can promote a transient change in the lactate/glucose ratio in inflamed cortical tissue. In this context, neuronal network activity can be maintained by metabolic adaptations including enhanced oxygen consumption. Indeed, lactate has been shown to increase oxygen consumption during network rhythms likely reflecting enhanced oxidative metabolism in mitochondria^9,24^.

Dynamic adaptations in brain metabolism and blood flow are indeed necessary to distribute energy substrates and oxygen to areas where neuronal activity is increased^3,4^. This process is important for synchronized network activities, such as gamma oscillations, which are exquisitely sensitive to disturbances in oxygen and energy substrate availability^2,41^. Our results suggest that the neuronal network can cope with moderate changes in the Lac/Glc ratio to sustain ATP production during gamma oscillations. This likely reflects the switch from some nonoxidative glycolysis and glycogenolysis in glucose only to oxidative metabolism in mitochondria in glucose plus lactate. This supplemental lactate oxidation seems to happen without any obvious side effects of intracellular acidification that might occur during H^+^-coupled lactate uptake^9^. By contrast, previous studies from our group showed that extreme alterations in lactate and glucose availability, i.e. provision of lactate in the absence of glucose, induce disturbances of gamma oscillations^24^. Notably, neuronal lactate uptake capacity was not expanded when we simulated high energy demand states, and, under low glucose availability, neurons presented energy deficit in all extracellular lactate concentrations (Fig. 4g–i). This might be a consequence of a limited lactate transport and conversion in neurons and agrees with evidence showing that proper neuronal stimulation requires glucose availability and a preserved glycolytic function^49–51^. Indeed, adding low fractions of glucose progressively suppressed neural bursting during gamma oscillations in hippocampal slices supplied with high lactate^24^.

Besides the alterations in lactate/glucose ratio, transient inflammation also associated with significant changes in Krebs cycle metabolites at the tissue level. Although the production of the immunometabolite itaconate could account for the interruption in the Krebs cycle flow during inflammation onset, the alterations in citrate and in the α-ketoglutarate/citrate ratio persisted after inflammation resolution (Fig. 1b, d; Supplementary Fig. 1a). This indicates that additional mechanisms, e.g., downregulation in the expression of isocitrate dehydrogenase, might mediate the reduced carbon flow through the Krebs cycle^52^. Of note, citrate and acetyl-CoA accumulation and the resulting changes in histone acetylation have been involved in inflammation progression and innate immune memory formation in myeloid cells^53–55^. This phenomenon raises the hypothesis that, although major inflammatory markers returned to control levels after resolution (Fig. 1b, c), microglia might still present an altered epigenetic landscape supporting modified responses upon secondary inflammatory stimulation. This hypothesis needs to be further investigated, however.

**Figure 5:**
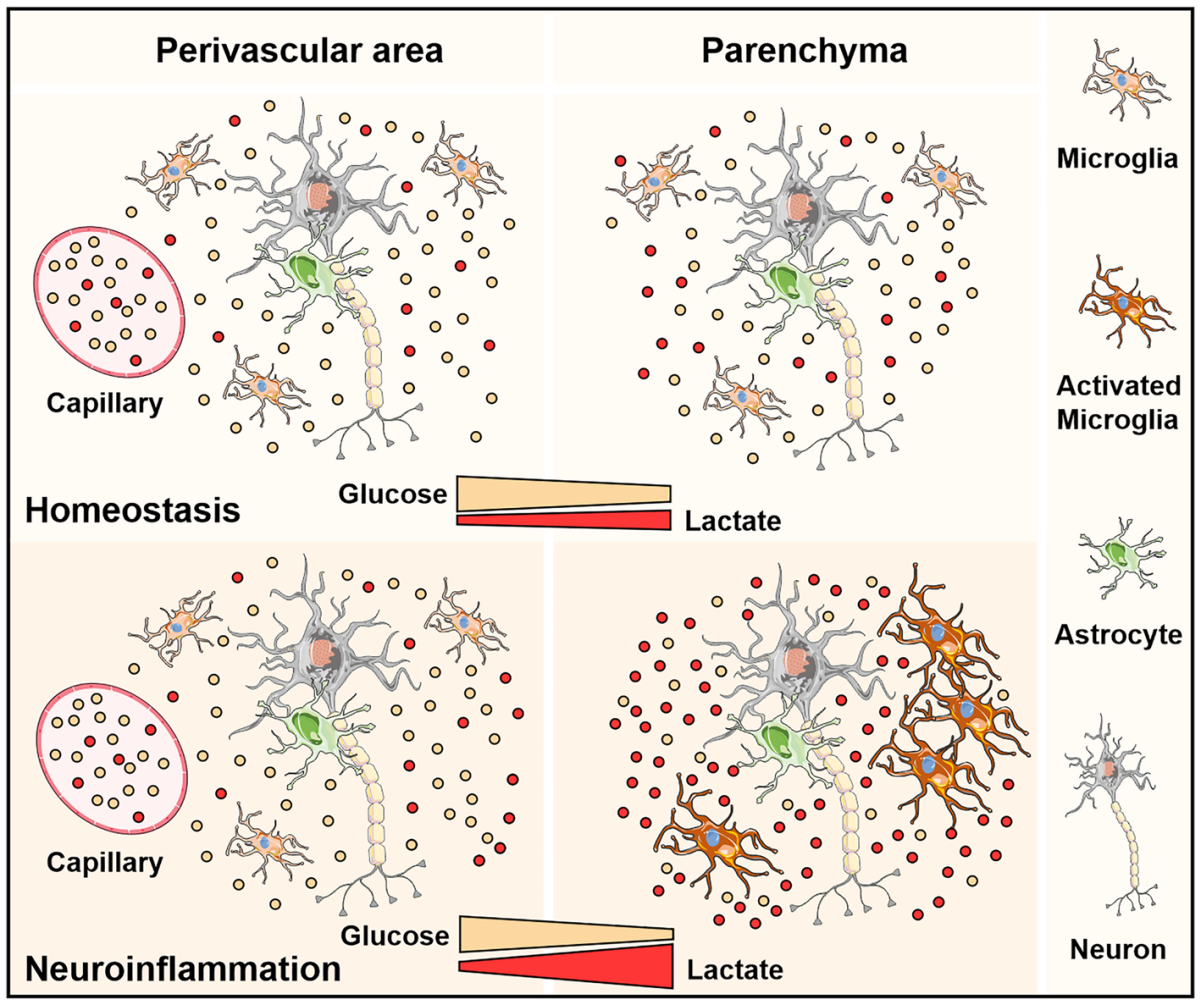
Metabolic flexibility ensures proper neuronal network function during inflammation. In homeostasis, the consumption of blood-imported glucose and the parallel production of lactate by brain cells generate a mild energy substrate gradient from the perivascular space, where the lactate/glucose ratio is low, to the parenchyma, where this ratio increases. During inflammation, microglial glucose consumption and lactate production are enhanced by a metabolic reprogramming, which results in an even higher lactate/glucose ratio, exacerbating the energy substrate gradient between the perivascular space and the brain parenchyma. In this scenario, the neuronal network activity might be preserved by a parallel increase in oxygen consumption. The boundaries for neuronal energy fitness during inflammation are high energy demand accompanied by extreme low glucose availability. These limits may be reached in niches of dysregulated inflammation, where clustering of reactive microglia may occur in areas farther from the capillaries.

The brain should continually face immunological challenges during a subject lifespan. Indeed, populations of activated microglia displaying proinflammatory molecular signature have been observed even in the healthy human brain^56^. In this context, the selection of a dynamic metabolic system represents an important evolutionary advantage ensuring accurate neuronal function while inflammation is solved. Of note, the consumption of blood-imported glucose and the local production of lactate should generate an energy substrate gradient from the perivascular space, where the Lac/Glc ratio is low, to the parenchyma, where this ratio increases (Fig. 5). During inflammation, microglia-mediated enhancement in glucose consumption and lactate production can result in an even higher Lac/Glc ratio, exacerbating the metabolic gradient between these two areas (Fig. 5). Although neurons can adapt to different metabolic compositions generated by microglial activation, it is attempting to speculate that the boundaries for neuronal metabolic flexibility could be reached in areas of dysregulated inflammation^9^. In fact, a recent study showed that microglial phenotypes are spatially distributed in human brain infection with the formation of clusters of reactive microglia in areas farther from the capillaries^57^. In this scenario, impairments in neuronal function and viability could be directly mediated by energy restriction resulting from alterations in microglial metabolism (Fig. 5).

Despite the evidence indicating that microglia may contribute to neuronal metabolic regulation^19–21^, upcoming studies should explore additional molecular interactions mediating cell communication in the brain. The molecular milieu in the inflamed brain is, in fact, much more complex than explored here and the production of inflammatory mediators can directly influence neuronal metabolism and function^23,26,27^. In niches of dysregulated inflammation, for instance, the release of oxidants such as nitric oxide can alter the structure and function of molecules and inhibit oxygen binding to respiratory complex IV in mitochondria, resulting in changes ranging from slowdown of neuronal network activity to severe neurodegeneration^27,30,58^.

Although our data suggest that metabolic changes during inflammation might be mostly driven by microglia, astrocytes can also contribute to the metabolic transitions in the inflamed brain. Indeed, microglia-astrocyte communication conveys inflammatory signals in diseases, which can be supported by a complex metabolic coupling between these cells^11,39,59^. Their individual contribution to these metabolic states needs to be further addressed. Furthermore, glucose hypometabolism has also been reported in distinct stages of neurodegenerative disorders and microglial transitions from a hyper- to hypometabolic state need to be further explored^21,60,61^.

We employed mathematical modeling to investigate the boundary conditions for changes in Lac/Glc ratio that active neurons can tolerate^43^. The use of a single neuron kinetic model, however, overrides the cellular diversity in the brain, where neuronal populations could be differentially susceptible to alterations in tissue substrate and oxygen availability^4,62^. In fact, while the extracellular oxygen concentration was kept constant in the model, it can vary in the brain depending on the metabolic state and the distance to the capillaries and, consequently, affect neuronal glucose and lactate consumption^4,43^. Mathematical modeling in tridimensional matrixes should be applied to further explore the metabolic constraints in the inflamed brain^43^.

In summary, our data suggest that moderate proinflammatory microglia can transiently alter substrate availability in the brain and induce adaptations in neuronal network fueling. Although neurons can cope with changes in substrate composition by adapting metabolism, high energy demand combined with low glucose availability may compromise neuronal survival and function in inflammatory niches. Further understanding of the spatiotemporal changes in cellular metabolism in the brain is expected to yield novel insights into the metabolic component of disorders featuring neuroinflammation.

## Methods

### Ethical statement

Rats were purchased from Janvier Laboratories (Le Genest-Saint-Isle, France) and handled in accordance with the European directive 2010/63/EU and with approval of the animal welfare officers at Heidelberg University (licenses, T-45/18 and T-37/21). Experiments were performed and reported in accordance with the ARRIVE guidelines.

### Animal handling, slice culture preparation and treatment

In this study, 9-day-old male Wistar rats were only employed in the preparation of organotypic hippocampal slice cultures. The pups were purchased from Janvier Laboratories and delivered at the age of 8-day-old (one day before slices preparation). There was no animal experimentation/treatment in this study. Note that the in vivo experiments reported in Fig. 2 were performed by Srinivasan et al. in their study published in Nature Communications, 2016. Here, we only performed additional analyses on the data sets deposited under the Gene Expression Omnibus GSE75246. See more details in the section RNA-sequencing analysis, Fig. 2 legend and results description.

Organotypic hippocampal slice cultures were prepared as previously described^30^. In brief, pups were quickly euthanized by decapitation without the use of anesthetics, brains were removed and hippocampal slices (400 µm) were cut with a McIlwain tissue chopper (Mickle Laboratory Engineering Company Ltd., Guildford, UK) under sterile conditions. Three slices with intact hippocampal structures were maintained on a Biopore™ membrane (Millicell standing inserts, Merck Millipore, Darmstadt, Germany) between culture medium, which consisted of 50% minimal essential medium, 25% Hank’s balanced salt solution (Sigma-Aldrich, Taufkirchen, Germany), 25% heat-inactivated horse serum (Life Technologies, Darmstadt, Germany), and 2 mM L-glutamine (Life Technologies) at pH 7.3, and humidified atmosphere with 5% (vol/vol) CO_2_ (36.5 °C) in an incubator (Heracell, Thermoscientific, Dreieich, Germany). Glucose concentration in the culture medium was about 4 mM, which was replaced three times per week. Experimentation started on day in vitro (DIV) 11. We note that slice cultures were maintained in the absence of antibiotics that have been reported to interfere with mitochondrial function in various cell types^63,64^. For pharmacological microglial depletion, liposome-encapsulated clodronate (Liposoma B.V., Amsterdam, The Netherlands) at a final concentration of 100 μg/mL was present in the culture medium from DIV 0 on and during the exposures^30^.

To explore tissue metabolic states during transient inflammation, membranes containing three cultured slice were exposed to 100 ng/mL LPS (from Escherichia coli, serotype R515; Alexis Biochemicals, via Enzo Life Sciences GmbH, Lörrach, Germany) (Fig. 1A). The membranes were randomly assigned to experimental groups. Medium of all membranes was daily changed. Six different groups were assigned (Table 1): “control”, exposed to medium only; “LPS”, exposed to four days medium and LPS in the last 24 h; “24 h”, exposed to three days medium, one day LPS and 24 h medium; “48 h”, exposed to two days medium, one day LPS and 48 h medium; “72 h”, exposed to one day medium, one day LPS and 72 h medium; and, “96 h”, exposed to LPS on the first day and then to 96 h medium. At the end of the fifth day, cultured slices from all groups were collected and frozen for RNA isolation and medium was frozen for biochemical analysis. Due to their return to a molecular profile similar to the control group, slices from the “96 h” treatment were referred to as resolution (RES) in later experiments.

**Table 1:** Experimental paradigm of transient inflammation in slice cultures (see also Fig. 1A). Medium was daily changed in all groups. LPS – lipopolysaccharide; RES—resolution.

Group/Day	1	2	3	4	5	6
Control	medium	medium	medium	medium	medium	**Tissue and medium collection**
LPS	medium	medium	medium	medium	LPS	
24 h RES	medium	medium	medium	LPS	medium	
48 h RES	medium	medium	LPS	medium	medium	
72 h RES	medium	LPS	medium	medium	medium	
96 h RES	LPS	medium	medium	medium	medium	

### RNA isolation and qRT-PCR

To determine the expression of target genes, six slice cultures pooled from two Biopore™ membranes were considered as a single sample for RNA isolation. RNA was isolated using RNeasy® Plus Mini kit (Qiagen, Hilden, Germany) followed by cDNA synthesis by High Capacity cDNA Reverse Transcription kit (Applied Biosystems, Foster City, CA, USA; via Life Technologies), both according to the manufacturer's instructions. The synthesized cDNA was used as a template for qPCR amplification carried by the StepOnePlus™ Real-Time PCR System (Applied Biosystems). Each PCR reaction contained 20 ng of cDNA, 200 nM of TaqMan assays (Life Technologies, see list in Table 2), TaqMan Fast Advanced Master Mix (Life Technologies) and ribonuclease-free water to a final volume of 10 μL. Reaction settings were 2 min at 50 °C, 2 min at 95 °C followed by 40 cycles of 1 s at 95 °C, 20 s at 60 °C. StepOnePlus™ software was used for comparative gene expression analysis, and β-actin was used as an endogenous control. Hierarchical clustering analysis is shown as heatmap and was visualized in Metaboanalyst using Euclidean and Ward.D as distance measure and clustering algorithm, respectively^65^. The color code represents then the number of standard deviations a sample is from the mean of expression distribution in the row. Negative z-scores indicate the value lies below the row mean and positive z-scores indicate the value lies above the row mean.

**Table 2:** Name and Taqman assay numbers of genes assessed in qRT-PCR experiments.

Gene	Gene Name	Taqman assay number
Microglial homeostatic genes		
P2RY12	Purinergic Receptor P2Y12	Rn02133262_s1
TMEM119	Transmembrane Protein 119	Rn01480631_m1
CX3CR1	C-X3-C Motif Chemokine Receptor 1	Rn02134446_s1
Inflammation- and stress-related genes		
NOS2	Nitric Oxide Synthase 2	Rn00561646_m1
IL1B	Interleukin 1 Beta	Rn00580432_m1
IL6	Interleukin 6	Rn01410330_m1
CD74	CD74 Molecule	Rn00565062_m1
TNF	Tumor Necrosis Factor	Rn99999017_m1
SOD2	Superoxide Dismutase 2	Rn00690588_g1
IL10	Interleukin 10	Rn99999012_m1
IRG1	Immune-Responsive Gene 1 Protein	Rn01467901_m1
IFNB1	Interferon Beta 1	Rn00569434_s1
ACTB	Actin Beta	Rn00667869_m1

### Cytokine detection

Enzyme-linked immunosorbent assays (ELISAs) were purchased from R&D (R&D Systems, Inc., Minneapolis, MN, USA; via Bio-Techne, Wiesbaden, Germany) and applied according to the supplier's protocol for the detection of interleukin-6 (IL-6; Cat. num. DY506). Briefly, capture antibodies were diluted in PBS (pH 7.2–7.4) and the reaction plate was coated overnight. The detection antibody for IL-6 was diluted in the reagent diluent, consisting of 1% bovine serum albumin in PBS (pH 7.2–7.4) supplemented with 2% normal goat serum. Eight-point standard curves were constructed from seven sequential two-fold dilution steps of recombinant IL-6 (8000 pg/mL) and the negative control consisted of non-treated culture medium. Samples were incubated in the coated reaction plate for 2 h. The detection antibody was then applied for 2 h and visualized with tetramethylbenzidine substrate solution (Moss Inc., Pasadena, USA). The development reaction was stopped with sulfuric acid, and the optical density was determined with a microplate reader (iMark, Bio-Rad GmbH, Munich, Germany) at 450 nm (with 540 nm reference). The concentrations of IL-6 were estimated by using the quadratic fit.

### Griess reaction

Nitric oxide release was estimated by determining nitrite levels using the Griess reaction carried out with undiluted culture medium. Nine-point standard curves were constructed by two-fold dilution steps of an 80 μΜ sodium nitrite standard (Merck Chemicals, Darmstadt, Germany). After addition of the Griess reagent mixture (0.05% 1-naphthylethylenediamine hydrochloride, 0.5% sulfanilamide and 2.5% orthophosphoric acid), the optical density was measured in a microplate reader at 540 nm (Bio-Rad). Nitrite concentration (μM) was calculated from the standard curve using linear fit.

### Determination of metabolites

Determination of organic acids was adapted from Uran et al.^66^. In brief, 6 cultured slices per sample were extracted in 0.2 mL ice-cold methanol with sonication on ice. Extracts were diluted with ultra-pure water 1:20 (vol/vol) and afterwards mixed with ice-cold methanol 1:4.5 (vol/vol). 50 µL of these extracts were mixed with 25 µL 140 mM 3-Nitrophenylhydrazine hydrochloride (Sigma-Aldrich), 25 µL methanol and 100 µL 50 mM Ethyl-3-(3-dimethylaminopropyl) carbodiimide hydrochloride (Sigma-Aldrich) and incubated for 20 min at 60 °C. Separation was carried out using an Acquity H-class UPLC system coupled to a QDa mass detector (Waters, Milford, MA, USA) using an Acquity HSS T3 column (100 mm × 2.1 mm, 1.8 µm, Waters) which was heated to 40 °C. Separation of derivates was achieved by increasing the concentration of 0.1% formic acid in acetonitrile (B) in 0.1% formic acid in water at 0.55 mL min^−1^ as follows: 2 min 15% B, 2 min 31% B, 5 min 54% B, 5.01 min 90% B, hold for 2 min, and return to 15% B for 2 min. Mass signals for the following compounds were detected in single ion record (SIR) mode using negative detector polarity and 0.8 kV capillary voltage: lactate (224.3 m/z; 25 V CV), itaconate (399.2 m/z; 15 V CV), malate (403.3 m/z; 25 V CV), succinate (387.3 m/z; 25 V CV), fumarate (385.3 m/z; 30 V CV), citrate (443.3 m/z; 10 V CV), pyruvate (357.3 m/z; 15 V CV) and α-ketoglutarate (550.2 m/z; 25 V CV).

### Lactate detection

Lactate concentration in culture supernatants was determined using the Amplite Colorimetric L-lactate assay kit (AAT Bioquest, Sunnyvale, CA, USA; via Biomol) according to the supplier's protocol. Briefly, each reaction contained 50 μL of lactate standards (1:2 serial dilution in PBS from 1000 μM stock solution, 6 points) or samples (1:5 dilution for slice culture medium in PBS) plus 50 μL L-lactate working solution (Enzyme probe and NAD^+^ in assay buffer). After 30 min incubation at room temperature, absorbance was read as the ratio 540 nm/595 nm. Lactate concentration was calculated from the standard curve using linear fit.

### Immunohistochemistry

Slice cultures were fixed in 4% paraformaldehyde in 0.1 M phosphate buffer for 2 h, incubated in 30% sucrose (AppliChem GmbH, Darmstadt, Germany) for additional 2 h and cut into 25 µm sections in a cryostat (CM1850; Leica Biosystems, Nussloch, Germany). Tissue was stained in free-floating sections. Unspecific immunoglobulin reactions were blocked with 5% normal goat serum (NGS) and slices were permeabilized with 0.3% Triton for 90 min. Sections were incubated with primary antibody (rabbit anti-Iba1, Fujifilm-WAKO Chemicals Europe GmbH, Neuss, Germany; 1:1000 in PBS with 10% NGS, 0.3% Triton and 0.1% NaN_3_) overnight. Next, sections were incubated with secondary antibody (Atto 488 anti-rabbit; 1:1000 in PBS (pH 6.8) with 5% NGS and 0.3% Triton) for 90 min. After washing steps with PBS (pH 6.8), sections were then incubated with DAPI (1:5000 in distilled water) for 5 min. Stained sections were placed on slides, dried and finally embedded with 30 µl of fluorescence mounting medium (DAKO, Agilent, Santa Clara, USA).

### Microglia counting

Confocal microscopy images were acquired in a Nikon C2 plus confocal microscope using the NIS-Elements software. Image acquisition was performed with a scan size of 2048 pixels and 1.2 pinhole using a Nikon Plan Apo 20 × objective. Confocal images were used to automatically count microglial cells using a macro in FIJI^67^. For counting, samples were randomized and analysis was performed blindly. Briefly, 5–7 image stacks (ca. 2.5 µm steps) were z-projected and the channels for DAPI and Iba1 were separated. FIJI’s *subtract background* function was employed to reduce noise in the Iba1 image. The DAPI image was converted to binary using FIJI’s *default dark* algorithm for automatic thresholding. A selection around the binary DAPI signal was created and overlayed on the Iba1 image to clear any signal not colocalizing with DAPI. The remaining Iba1 signal was converted to binary using FIJI’s *default dark* algorithm. Noise was further reduced by the *despeckle* and *remove outlier* functions. Remaining binary shapes were automatically counted by FIJI’s *analyze particles* function with a cutoff value of 400 pixels to exclude areas where nuclei of other cell types colocalized with microglial ramifications.

### RNA-sequencing analysis

RNA sequencing data were generated by Srinivasan et al. 2016, which are deposited in NCBI’s Gene Expression Omnibus under the accession number GSE75246^37^. In their experimental design, mouse microglia, neurons and astrocytes were separated by cell sorting after 24 h intraperitoneal LPS injection and were then employed in bulk RNA-seq analyses. We uploaded raw count data into iDEP (Integrated Differential Expression and Pathway Analysis – v 0.95, accession dates: Feb 10th, 2022, and Mar 09th, 2023)^68^. Differentially expressed genes were analyzed using DEseq2, with cut-offs of fold change (FC) > 2 and false discovery rate (FDR) of < 0.1. Differential expression analysis was first performed in whole genome comparing LPS versus PBS microglia and LPS versus PBS astrocytes. We next focused on changes in metabolism by performing differential expression analyses on genes involved in carbohydrate metabolic processes, ATP processes, TCA processes, and individually added metabolic regulators and transporters (complete list in supplementary information) and compared LPS vs PBS microglia, LPS versus PBS astrocytes and LPS versus PBS neurons. Scatter and Volcano plots were generated in iDEP. A fold change of 2 was used as cutoff for inclusion in the analysis of co-modulation in metabolic genes presented in Supplementary Fig. 4a–c. A database published in Friedman et al.^69^ were used to collect and analyze expression data present in Supplementary Fig. 4.

### Electrophysiology

For electrophysiological recordings, the Biopore™ membrane carrying slice cultures was inserted into the recording chamber^30^. Slice cultures were maintained at the interface between recording solution (artificial cerebrospinal fluid (ACSF), rate 1.8 mL/min) and ambient gas mixture (20% O_2_, 5% CO_2_ and 75% N_2_, rate 1.5 L/min), which permits constant oxygen supply to the tissue. ACSF contained 129 mM NaCl, 3 mM KCl, 1.25 mM NaH_2_PO_4_, 1.8 mM MgSO_4_, 1.6 mM CaCl_2_, 21 mM NaHCO_3_ and 5 mM glucose. The pH was 7.3 when the recording solution was saturated with the gas mixture. Recordings were done at 34 ± 1 °C. Gamma oscillations were elicited by continuous application of 10 µM carbachol via the recording solution. Such cholinergic gamma oscillations in situ share many features with gamma oscillations in vivo and require both neuronal excitation and fast inhibition^2,31,40^. Standard salts and carbachol were purchased from Sigma-Aldrich.

For experiments with changes and energy substrate availability, gamma oscillations were elicited in untreated slice cultures by continuous application of 10 µM carbachol in ACSF containing 5 mM glucose saturated with gas mixture (20% O_2_, 5% CO_2_ and 75% N_2_). After oscillation signal was stable (35 min), ACSF containing 5 mM glucose plus 2 mM lactate was perfused in the interface chamber and recordings were performed for additional 15 min. Since it lasts 8 min until the recording solution achieves the interface chamber, 3 min recordings were analyzed at 40–43 min for the pre-washing and 45–48 for the post-washing condition. Gamma oscillations were characterized in both directions (Glc → Glc + Lac, or Glc + Lac → Glc) to exclude a time contribution to the results.

Local field potentials (LFP) were recorded with glass electrodes (resistance of 1–2 MOhm) that were made from GB150F-8P borosilicate filaments (Science Products GmbH, Hofheim, Germany) filled with ACSF. The electrode was positioned in the stratum pyramidale of the CA3 region with a mechanical micromanipulator (MM 33, Märzhäuser, Wetzlar, Germany). LFP were recorded with an EXT 10-2F amplifier in EPMS-07 housing (npi electronic GmbH, Tamm, Germany), low-pass filtered at 3 kHz, and digitized at 10 kHz using CED 1401 interface and Spike2 software (Cambridge Electronic Design, Cambridge, UK) for offline analysis.

### Oxygen profiling in slice cultures

Oxygen concentration was measured at slice cores of the CA3 area by using oxygen sensor microelectrodes, standard OX10 (Unisense A/S, Aarhus, Denmark). This modified polarographic Clark electrode consists of a glass-insulated Ag/AgCl reference anode and it has a tip diameter of 10 µm and a spatial resolution of 1–2 times the outside tip diameter. The O_2_ sensor was connected to a 4-channel microsensor multimeter (Unisense A/S) and polarized with − 0.8 V overnight. For recordings, the O_2_ sensor was fixed in a mechanical micromanipulator at an angle of 55° and moved forward in steps of 20 µm (corresponding to a vertical depth of ~ 16 µm per step). Before and after each experiment, O_2_ sensors were individually calibrated using a three-point calibration with ACSF saturated with 0%, 20% and 95% O_2_, respectively^70^. Slice cores were determined as the point presenting the lowest O_2_ concentration during tissue profiling. Note that decreases in O_2_ concentration in slice cores represent increased oxygen consumption at the tissue level.

### Metabolic model

Neuronal energy states in different Lac/Glc ratios were simulated using a kinetic model of single neuron energy metabolism^43^. The model describes the molecular resolved central ATP-producing pathways. It distinguishes the cytosolic and mitochondrial compartment and comprises glycolysis, the citric acid cycle, the respiratory chain, oxidative phosphorylation, mitochondrial electrophysiology (including mitochondrial calcium dynamics) as well as the malate-aspartate shuttle and the glycerol-3-phosphate shuttle, coupling the cytosolic and mitochondrial NAD/NADH pools. It describes the exchange of glucose and lactate as well as oxygen with the extracellular compartment. Kinetic rate equations for the individual enzymes were constructed on the basis of kinetic data.

Energy states were determined by systematically varying glucose availability, lactate availability and energy demand and by monitoring glucose uptake, lactate uptake/release as well as intracellular ATP as a proxy of neuronal energy fitness. Extracellular oxygen concentration was kept constant at saturating levels of 60 mmHg. Alterations in energy demand were simulated by modeling ATPase activity. ATP consumption was modelled by v_ATPase = k*ATP/(ATP + Km) and increased energy demand was modeled by increasing the rate constant k. The biochemical and biophysical processes as well as all equations included in the kinetic model are described in detail in Berndt et al., 2015^43^.

### Data analysis and statistics

Offline signal analysis of gamma oscillations was performed in MatLab R2017b (The MathWorks, Inc., Natick, MA, USA) using scripts developed and kindly supplied by Dr. Jan-Oliver Hollnagel^13,24^. Data segments of 3 min or 5 min were low-pass filtered with a digital Butterworth algorithm at 200 Hz corner frequency and processed with Welch's algorithm with a Hamming window size of 8192 points for calculation of the power spectral density (bin size = 1.2207 Hz). Gamma oscillations were analyzed for various parameters, i.e., peak frequency (frequency), peak power (power), and full width at half-maximum (FWHM). Electrical network activity was classified as gamma oscillation if the frequency was higher than 24 Hz at 34 ± 1 °C and the peak power higher than 10^–4^ mV^2^/Hz^31^.

Data are presented as mean ± SEM derived from independent experiments representing different preparations of rat pups, unless stated otherwise. Statistical significance (*P* < 0.05) was determined in GraphPad Prism® 8.0 (GraphPad Software, California, USA). Data distribution was tested for normality with the Shapiro–Wilk test. Statistical tests are specified in the figure legends. Figures were created with GraphPad Prism® 8.0 (GraphPad Software), and CorelDRAW (Corel, Ottawa, Ontario, Canada).

### Supplementary Information

Supplementary Information 1.

Supplementary Information 2.

## Data Availability

The datasets generated during and/or analyzed during the current study are available from the corresponding author on reasonable request.
