# Supplementary material for: Metabolic flexibility ensures proper neuronal network function in moderate neuroinflammation
Source: Sci Rep. 2024 Jun 22;14:14405. doi: 10.1038/s41598-024-64872-1 (PMC11193723; doi:10.1038/s41598-024-64872-1)
Supplement: Supplementary file 1 — Supplementary Information 1. [file 41598_2024_64872_MOESM1_ESM.pdf]

Supplementary figures

Metabolic flexibility ensures proper neuronal network function in moderate neuroinflammation

Bruno Chausse[1,7*], Nikolai Malorny[1], Andrea Lewen[1], Gernot Poschet[2], Nikolaus Berndt[3,4,5], Oliver Kann[1,6*]

Affiliation: [1]Institute of Physiology and Pathophysiology, Heidelberg University, D-69120 Heidelberg, Germany. [2]Metabolomics Core Technology Platform, Centre for Organismal Studies, Heidelberg University, D-69120 Heidelberg, Germany. [3]Department of Molecular Toxicology, German Institute of Human Nutrition Potsdam-Rehbruecke (DIfE), D-14558 Nuthetal, Germany. [4]Institute of Computer-assisted Cardiovascular Medicine, Deutsches Herzzentrum der Charité (DHZC), D-13353 Berlin, Germany. [5]Charité – Universitätsmedizin Berlin, corporate member of Freie Universität Berlin and Humboldt-Universität zu Berlin, D-10117 Berlin, Germany. [6]Interdisciplinary Center for Neurosciences (IZN), Heidelberg University, D-69120 Heidelberg, Germany. [7]Current Address: MEDISS Doctoral Program, INF 110, Heidelberg University, D-69120 Heidelberg, Germany.

***Corresponding authors:**

Dr. Bruno Chausse, MEDISS Doctoral Program, INF 110, Heidelberg University, D-69120 Heidelberg, Germany; Phone: 0049(0)6221.5635977 E-mail: bruno.chaussedefreitas@med.uni-heidelberg.de

Dr. Oliver Kann, Institute of Physiology and Pathophysiology, Heidelberg University, Im Neuenheimer Feld 326, D-69120 Heidelberg, Germany; Phone: 0049(0)6221.544560 E-mail: oliver.kann@physiologie.uni-heidelberg.de

This file contains the supplementary figures 1-4.

Supplementary Figure 1

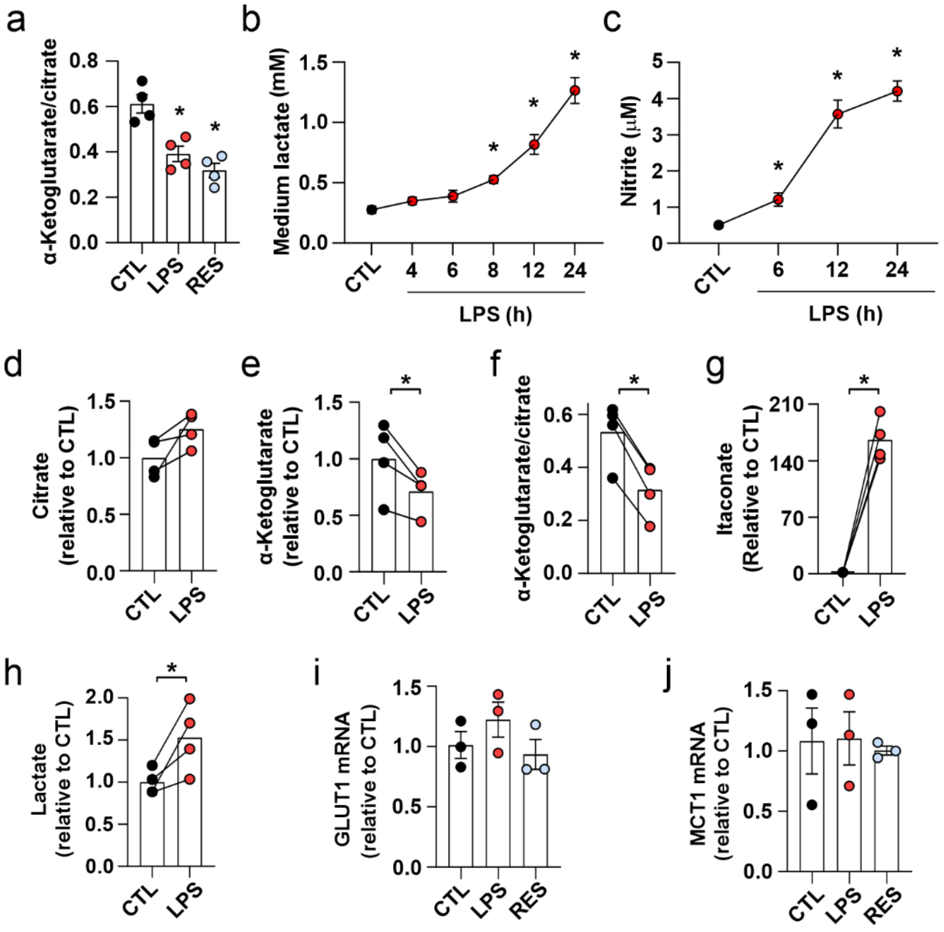

Supplementary Figure 1: Moderate inflammation transiently alters brain energy metabolism. (**a,i,j**) Slice cultures were exposed to medium (Control group - CTL), LPS (100 ng/mL) in the last 24 h before tissue processing (LPS group) or LPS for 24 h followed by incubation with medium only for additional 96 h (Resolution group - RES). Medium from all groups was changed daily. (**a**) α-ketoglutarate/citrate ratio in whole tissue homogenates. (**b-h**) Slice cultures were exposed to medium (CTL) or LPS (100 ng/mL) in the indicated time-course (**b,c**) or for 8 hours (**d-h**). (**b**) Lactate and (**c**) nitrite were measured in culture supernatants. (**d**) Citrate, (**e**) α-ketoglutarate, (**f**) α-ketoglutarate/citrate ratio, (**g**) itaconate and (**h**) lactate content in whole tissue homogenates. (**i**) GLUT1 and (**j**) MCT1 mRNA expression in tissue homogenates. Except in **b** and **c**, values are normalized by the content in the control group. Values represent averages ± SEM and were compared using paired t test (**d-h**), one-way ANOVA followed by Tukey's post hoc test (**a,i,j**) or one-way ANOVA followed by Dunnett's post hoc test (**b,c**). *$P < 0.05$ vs CTL. For n/N membranes/animals: (**a,d-h**) 4 pools/8 animals, (**b,c**) 4-20 membranes/4-20 animals, (i,**j**) 3 pools/6 animals.

Supplementary Figure 2

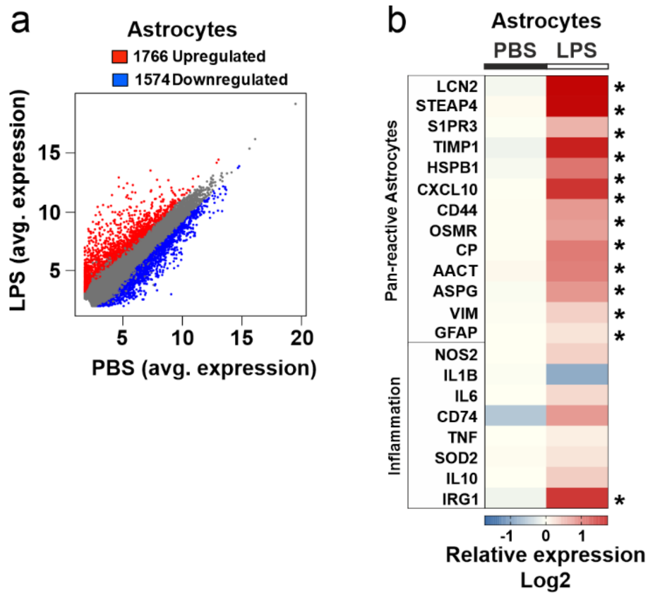

Supplementary Figure 2: A transcriptional reprogramming in astrocytes during brain inflammation in vivo. The dataset generated by Srinivasan et al. 2016 (GSE75246) was employed to explore cell-specific changes in metabolic genes during inflammation. In their experimental design, microglia, neurons and astrocytes were separated by cell sorting 24 h after intraperitoneal LPS injection (ipLPS) and then characterized by bulk RNAseq analyses. (**a**) Scatter plot showing differentially expressed genes in ipLPS versus ipPBS (intraperitoneal PBS injection) astrocytes (fold change > 2; false discovery rate cutoff: 0.1; see Methods). Note that intraperitoneal LPS effectively induces a transcriptional reprograming in astrocytes. (**b**) Differential expression of panreactive-astrocytes and inflammatory genes in ipLPS versus ipPBS astrocytes. Heatmap color code represents medians of the relative gene expression (Log2). Note that the inflammatory genes changing in ipLPS astrocytes almost not overlay with the direct exposure of slice cultures to LPS (**Fig. 1b**). *Padj < 0.05. N/group: 4 animals/treatment group.

Supplementary Figure 3

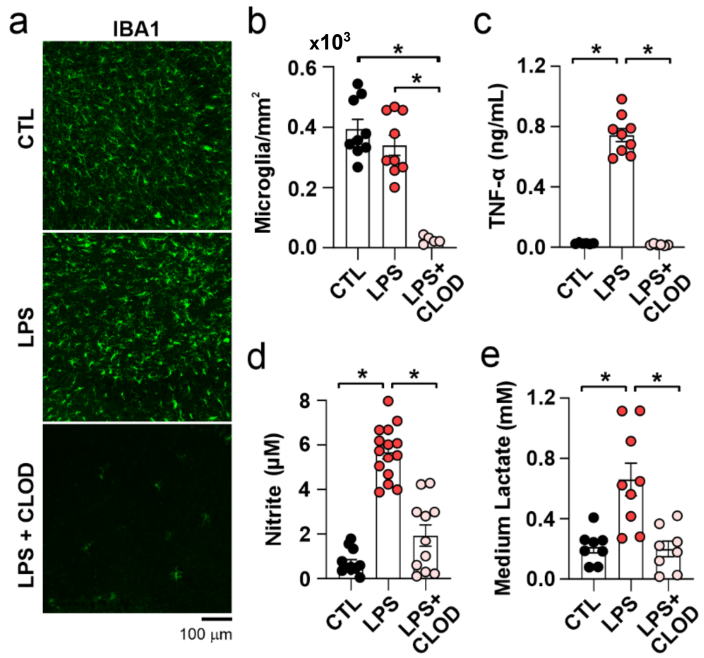

Supplementary Figure 3: Microglial depletion prevents LPS-induced inflammatory and metabolic changes in slice cultures. Naive and microglia-depleted hippocampal slice cultures were exposed to 100 ng/ml LPS for 24 h. (**a**) Representative images of slices stained against the microglia-specific marker Iba1. (**b**) Quantification of Iba1-positive cells in hippocampal stratum radiatum. (**c**) TNF-α (**d**) nitrite and (**e**) lactate content in culture supernatants. Values represent averages ± SEM and were compared using one-way ANOVA followed by Tukey's post hoc test (**b**,**c**,**e**) or Kruskal-Wallis test followed by Dunn's post hoc test (**d**). *$P < 0.05$. For n/N membranes/animals: 6-15/6-15.

Supplementary Figure 4

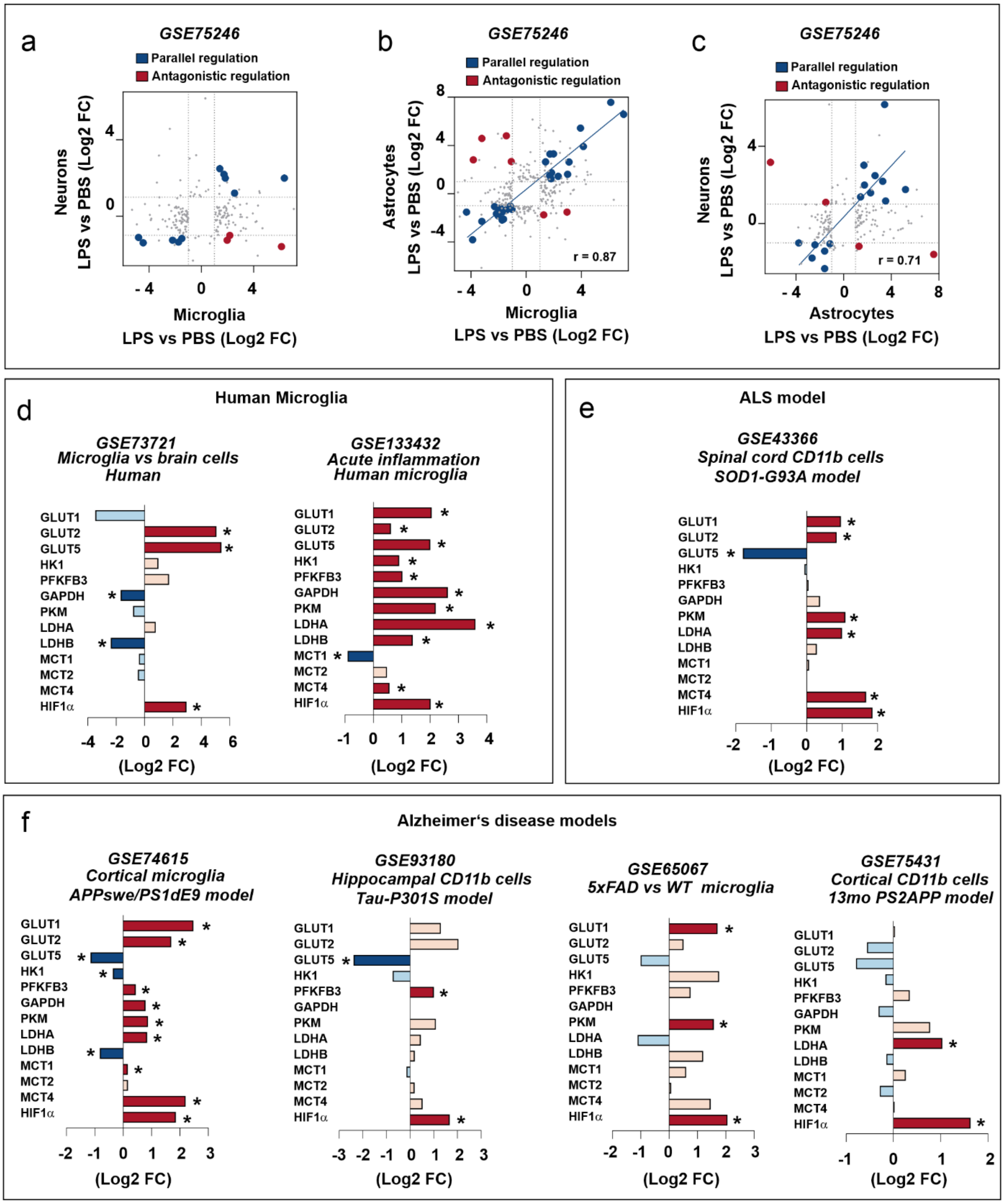

Supplementary Figure 4: A transcriptional reprogramming in microglia drives the alterations in brain metabolism during inflammation. The dataset generated by Srinivasan et al. 2016 (GSE75246) was employed to explore cell-specific changes in metabolic genes during inflammation. In their experimental design, microglia, neurons and astrocytes were separated by cell sorting after 24 h intraperitoneal LPS injection and were then employed in bulk RNAseq analyses. Parallel (blue) and antagonistic (red) regulation of metabolic gene expression in (**a**) microglia versus neurons, (**b**) microglia versus astrocytes (parallel regulation: Spearman r 0.8719; P <

0.0001) and (**c**) astrocytes versus neurons (parallel regulation: Spearman r 0.7071; P = 0.0042). N/group: For microglia and neurons: 5 animals/treatment group, for astrocytes: 4 animals/treatment group. A database published in Friedman et al. 2018 was used to collect and analyze the expression of microglial glycolytic genes in (**d**) engrafted human microglia, (**e**) amyotrophic lateral sclerosis (ALS), and (**f**) Alzheimer's disease models. NCBI's Gene Expression Omnibus accession numbers are displayed in the figure. FC – Fold Change. *Padj < 0.05.
